# Apigenin, Selenium, and Combinations Are Effective in Preventing 5‐Fluorouracil Induced Cardiovascular Damage

**DOI:** 10.1002/jbt.71028

**Published:** 2026-07-30

**Authors:** Gözde Atila Uslu, Hamit Uslu, Taha Abdulkadir Çoban, Emine Toraman, Mustafa Özkaraca

**Affiliations:** ^1^ Department of Physiology Faculty of Medicine, Erzincan Binali Yıldırım University Erzincan Türkiye; ^2^ Department of Biochemistry Faculty of Medicine, Erzincan Binali Yıldırım University Erzincan Türkiye; ^3^ Department of Molecular Biology and Genetics Science Faculty, Atatürk University Erzurum Türkiye; ^4^ Department of Pathology Faculty of Veterinary Medicine, Cumhuriyet University Sivas Türkiye

**Keywords:** 5‐fluorouracil, apigenin, apoptosis, cardiovascular damage, oxidative stress, selenium

## Abstract

Although chemotherapy is still a widely used primary treatment modality for many cancers, it is an accepted fact that it causes many undesirable side effects and toxicity. Until a new, side‐effect‐free treatment modality is developed, new resources will continue to be sought to prevent and reduce the adverse effects of chemotherapy. In this study, we investigated the potential of apigenin, selenium, and their combination to prevent cardiovascular toxicity induced by 5‐fluorouracil (5FU), an important chemotherapeutic agent. The study consisted of control (C), 5FU, apigenin + 5FU (Api + 5FU), selenium + 5FU (Se + 5FU), and apigenin + selenium + 5FU (Api + Se + 5FU) groups. Api (50 mg/kg) and Se (0.5 mg/kg) were administered orally to rats for 7 days. On Day 5, 5FU was administered i.p. at a dose of 150 mg/kg. Administration of 5FU significantly reduced GSH levels and CAT and TrxR activities in both cardiac and thoracic aortic tissues. It also decreased Bcl‐2 expression and increased Bax and Caspase‐3 expression in cardiac tissue, while reducing Bcl‐2 and increasing Bax expression in the thoracic aorta. Thoracic aortic MDA levels were also significantly elevated. On the contrary, oxidative stress and apoptosis were significantly decreased, and cardiovascular damage was attenuated in apigenin and selenium treated groups. In this study, it was determined that 5FU caused a higher level of toxicity to the heart compared to the thoracic aorta. In addition, both apigenin and selenium were shown to have significant protective effects on the cardiovascular system against 5FU toxicity. However, the cardiovascular protection against 5FU toxicity was found to be higher when apigenin and selenium were used together.

## Graphical abstract

5‐Fluorouracil (5‐FU) induces cardiovascular toxicity by increasing ROS, MDA, Bax, and caspase‐3 while decreasing antioxidant defenses and Bcl‐2 levels. Apigenin and selenium enhance antioxidant capacity, restore Bcl‐2 expression, suppress apoptosis, and rebalance redox status. The combination of these two provides more effective protection against 5‐FU‐induced heart and aortic damage.

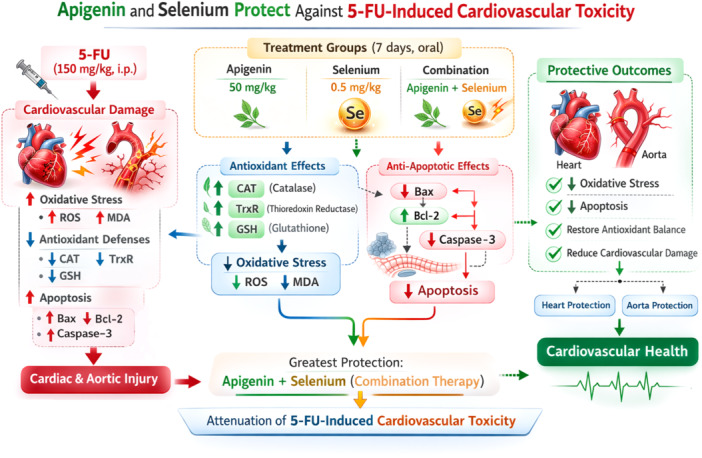

## 1 Introduction

Chemotherapy is one of the most widely used anticancer strategies, aimed at inhibiting tumor growth and progression through the administration of cytotoxic agents. Although it can improve survival and disease control depending on disease stage and patient‐related factors, its clinical use is often limited by systemic adverse effects that negatively impact quality of life [1].

5‐Fluorouracil (5‐FU) is a widely used antimetabolite chemotherapeutic agent that has been employed either alone or in combination regimens for the treatment of various malignancies, including breast, prostate, colorectal, bladder, pancreatic, and head and neck cancers [2, 3]. Its antitumour activity is primarily mediated through inhibition of thymidylate synthase (TS), following intracellular conversion to its active metabolite fluorodeoxyuridine monophosphate (FdUMP), which leads to impaired DNA synthesis via formation of a ternary complex involving FdUMP, TS, and 5,10‐methylene tetrahydrofolate (CH_2_THF). In addition, 5‐FU disrupts RNA processing and function [4]. Despite its therapeutic efficacy, its non‐selective cytotoxicity toward normal tissues and the development of chemoresistance—mediated by alterations in drug targets, activation of DNA repair pathways, and evasion of apoptosis—remain major limitations in clinical practice [5, 6].

Among the organ systems affected, the cardiovascular system has been increasingly recognized as a critical target of 5‐FU‐induced toxicity. Clinical evidence indicates that cardiotoxicity occurs in approximately 1.2%–7.6% of patients receiving 5‐FU and represents a clinically relevant complication of therapy [7, 8]. Although the underlying mechanisms are not fully understood, reported manifestations include arrhythmias, myocardial infarction, dyspnea, angina pectoris, left ventricular dysfunction, cardiac arrest, and sudden death [8, 9]. Experimental studies have further demonstrated that 5‐FU induces oxidative stress, inflammation, and myocardial injury, as evidenced by elevated cardiac injury markers and malondialdehyde (MDA) levels, together with reduced antioxidant capacity and histopathological damage [10, 11].

Natural compounds have long been considered valuable sources for the development of novel therapeutic agents. Apigenin (4′,5,7‐trihydroxyflavone), a naturally occurring dietary flavonoid, has attracted considerable attention due to its antioxidant, anti‐inflammatory, and antineoplastic properties [12, 13, 14]. In spontaneously hypertensive rats, infusion of apigenin into the hypothalamic paraventricular nucleus reduced mean arterial pressure, heart rate, plasma norepinephrine levels, cardiac hypertrophy, perivascular fibrosis, oxidative stress, and inflammation, indicating a potential cardioprotective effect of apigenin [15]. Consistent with these findings, apigenin also exerted cardioprotective effects in ischemia‐reperfusion models by reducing myocardial injury, infarct size, and apoptosis, accompanied by decreased Bax and caspase‐3 expression and increased Bcl‐2 expression [16].

Selenium is an essential trace element involved in multiple physiological processes, including antioxidant defense, thyroid hormone metabolism, and reproductive function [17]. Experimental evidence suggests that selenium supplementation enhances antioxidant enzyme activity, including superoxide dismutase (SOD), reduced glutathione (GSH), and glutathione peroxidase (GPx), while improving cardiac structure and function in models of cardiac injury [18]. Moreover, selenium has been reported to exert cardioprotective effects by reducing oxidative stress, modulating gene expression, and inhibiting inflammatory signaling pathways [19, 20].

In light of these findings, the present study aimed to investigate the protective effects of apigenin and selenium, administered alone or in combination, against 5‐FU‐induced cardiac and thoracic aortic injury, with particular emphasis on oxidative stress and apoptosis.

## 2 Materials and Methods

### 2.1 Animal Model

Thirty‐five male Sprague‐Dawley rats weighing 203 ± 10 g were used in this investigation. Erzincan Binali Yıldırım University Experimental Animal Research and Application Center provided the rats, which were kept at a standard temperature of 25°C with a 12‐h light‐dark cycle, sufficient ventilation, and free access to a regular feed and water. The body weights of the rats were determined before starting the experiment and randomly divided into five groups. Control group (C), 5FU group, apigenin + 5FU (Api + 5FU) group, selenium + 5FU (Se + 5FU) group, and the apigenin + selenium + 5FU (Api + Se + 5FU) group. Selenium was administered at a dose of 0.5 mg/kg and apigenin at a dose of 50 mg/kg orally for 7 days, and 5FU was administered as a 150 mg/kg single dose i.p. to all groups except the C group on the 5th day of the study. Saline was applied to this group in order to minimize the stress that may develop due to substance application between the control group and the substance‐treated groups. Heart and thoracic aorta samples were taken from euthanized animals on the 8th day of the study.

### 2.2 Biochemical Analyses

Heart and thoracic aorta samples from rats in all groups were washed with PBS and homogenized using phosphate buffer [21]. The protein amounts of the supernatants formed by centrifugation of the homogenate were determined according to the Bradford method and used for analysis [22].

### 2.3 Determination of GSH Levels

The Sedlak and Lindsay method was used to measure GSH levels. The supernatant obtained from homogenized heart and thoracic aorta samples was used to measure GSH (mM) with 5,5′‐dithiobis‐(2‐nitrobenzoic acid). GSH content was determined by spectrophotometric absorbance measurement at 412 nm [23].

### 2.4 Determination of MDA Levels

The thiobarbituric acid (TBA) assay was used to determine lipid peroxidation (LPO) levels in rat heart and thoracic aorta homogenates [24]. MDA reacts with TBA to form a pink‐colored product in this test. The amount of MDA was determined by spectrophotometric absorbance measurement at 532 nm [25].

### 2.5 Determination of CAT and Thioredoxin Reductases (TrxR) Enzyme Activities

The enzymatic activities of CAT and TrxR in rat heart and thoracic aortic tissue homogenates were measured according to the methods described by Aebi and Hill, respectively [26, 27].

### 2.6 Histopathological Evaluations

Some of the samples from the aorta and heart were preserved in a 10% neutral formalin solution. After undergoing a standard alcohol‐xylol process, the tissues were put in paraffin blocks. 5 µm thick serial sections were stained with haematoxylin‐eosin. The sections were evaluated semiquantitatively for degenerative and inflammatory changes as absent (−), mild (+), moderate (++), and severe (+++) under a light microscope. For immunohistochemical evaluations, 5 μm sections were taken onto polylysine slides and washed with PBS after passing through xylol and alcohol series and then kept in 3% H_2_O_2_ for 10 min to ensure inactivation of endogenous peroxidase. Antigen retrieval solution was used to reveal the antigen in the tissues and treated at 500 watts for 2 × 5 min. After protein block, the tissues were washed with PBS and incubated with Caspase‐3 (Elabscience, Catalog no. E‐AB‐30004), Bax (Elabscience, Catalog no. E‐AB‐13814), and Bcl‐2 (Elabscience, Catalog no. E‐AB‐60012) primary antibodies at a dilution ratio of 1/200 at +4°C overnight. As a secondary large volume detection system, anti‐polyvalent HRP (Thermofischer, Catalog No. TP‐125‐HL) was used as recommended by the manufacturer. DAB (3,3′‐Diaminobenzidine) was used as a chromogen. After counterstaining with Mayer's haematoxylin, they were covered with aqueous mounting medium and examined under a light microscope. Immunopositivity was evaluated semiquantitatively as absent (−), mild (+), moderate (++), and severe (+++).

### 2.7 Statistical Analyses

Statistical analysis of all biochemical measurements was performed using GraphPad Prism software version 9.0 (GraphPad Software, San Diego, CA). Statistical comparisons were made by one‐way ANOVA tests. The obtained histological and immunohistochemical data were analyzed with the SPSS 20.00 program. The Kruskal‐Wallis nonparametric test was used to evaluate the difference between the groups, while the Mann‐Whitney *U* test was used to identify the group responsible for the difference (*p* < 0.05).

## 3 Results

### 3.1 Body Weight Changes

**Figure 1:**
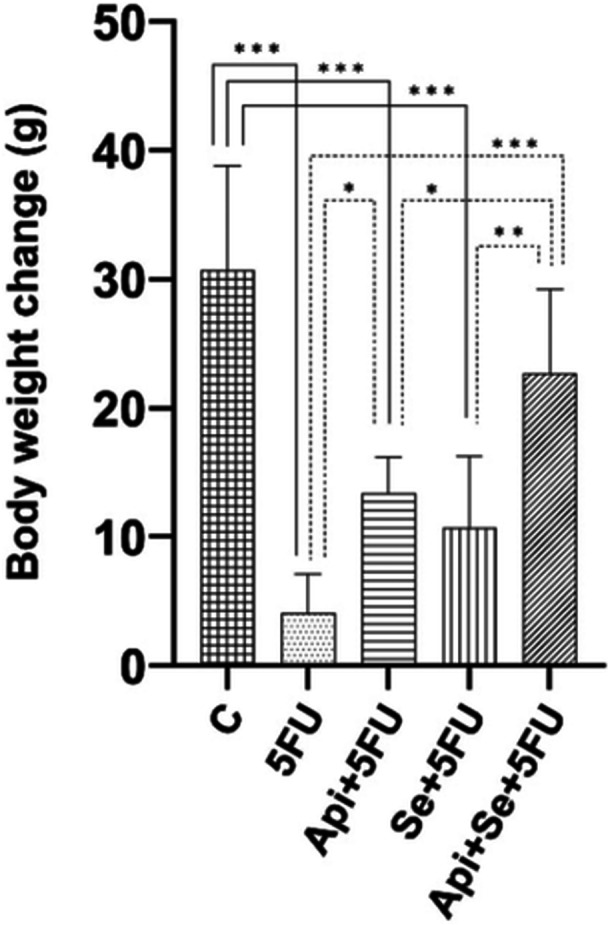
Body weight changes (g) of animals in control and experimental groups. *p < 0.05, **p < 0.01, ***p < 0.001.

At the beginning of the study, the body weights of the animals were similar to each other and there was no statistical difference between the groups. However, at the end of the study, it was determined that there were significant differences in body weight changes. It was observed that the animals in the C group continued their normal development, whereas the development of the animals in the 5FU group was inhibited and accordingly, their body weights remained significantly lower (*p* < 0.001). The changes in body weights of the Api + 5FU, Se + 5FU, and Api + Se + 5FU groups were higher than the 5FU group. However, these changes were significant only in the Api + 5FU (*p* < 0.05) and Api + Se + 5FU (*p* < 0.001) groups. It was remarkable that the change in the Api + Se + 5FU group was similar to the C group (Figure 1).

### 3.2 Histopathological Results

**Figure 2:**
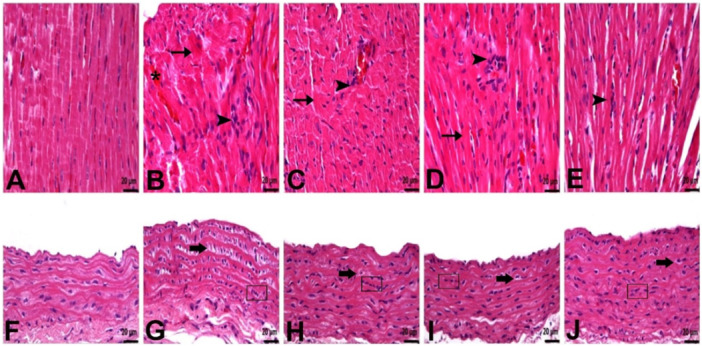
(A–E) Histopathological findings in myocardium (A) Control group. Normal histological appearance, (B) 5FU group. Severe MN cell infiltrations (arrowhead) and degeneration (arrow) and moderate hemorrhage (*), (C) Api + 5FU group. Mild degeneration (arrow) with moderate MN cell infiltrations (arrowhead), (D) Se + 5FU group. Moderate MN cell infiltrations (arrowhead) with degeneration (arrow), (E) Api + Se + 5FU group. Mild MN cell infiltrations (arrowhead), (F–J) Histopathological findings in the aorta (F) Control group. Normal histological appearance, (G) 5FU group. Severe degeneration of smooth muscle cells (arrow) with pyknosis (□), (H) Api + 5FU group, (I) Se + 5FU group, and (J) Api + Se + 5FU group. Mild degeneration of smooth muscle cells (arrow) and pyknosis (□), (H‐E) MN: Mononuclear cell infiltration.

Significant differences were found between the groups in terms of histopathological findings in the heart and thoracic aorta. Histopathological findings included mononuclear cell infiltration in the myocardium and degeneration and hemorrhage in myocytes. Mononuclear cell infiltrations were severe in the 5FU group, moderate in the Api + 5FU and Se + 5FU groups, and mild in the Api + Se + 5FU group. Myocyte degeneration was severe in the 5FU group, moderate in the Se + 5FU group, mild in the Api + 5FU group, and nonsignificant in the Api + Se + 5FU group. Only the 5FU group experienced moderate hemorrhage (Table 1, Figure 2). Microscopic alterations were found in the aorta's tunica media layer. While the 5FU group showed significant degeneration and pyknotic alterations in smooth muscle cells in the tunica media, the other groups had milder histological results (Table 2, Figure 2).

**Table 1:** Statistical evaluation of histopathological findings in myocardium.

Groups	Mononuclear cell infiltration	Myocyte degeneration	Myocardial hemorrhage
**C**	0.33 ± 0.52^a^	0.33 ± 0.52^a^	0.00 ± 0.00^a^
**5FU**	2.83 ± 0.41^b^	2.67 ± 0.52^b^	1.50 ± 0.55^b^
**Api** + **5FU**	2.00 ± 0.00^c^	1.33 ± 0.52^e^	0.00 ± 0.00^a^
**Se** + **5FU**	2.16 ± 0.41 ^cd^	1.83 ± 0.41^de^	0.00 ± 0.00^a^
**Api** + **Se** + **5FU**	1.17 ± 0.41^e^	0.33 ± 0.52^a^	0.00 ± 0.00^a^

*Note:* a‐b, a‐c, a‐cd, a‐de, b‐c, b‐e, c‐e, cd‐e: *p *< 0.01; a‐e, b‐cd, b‐de: *p *< 0.05.

**Table 2:** Statistical evaluation of histopathological findings in thoracic aorta.

Groups	Degeneration	Pyknosis
**C**	0.00 ± 0.00^a^	0.17 ± 0.41^a^
**5FU**	2.67 ± 0.52^b^	2.83 ± 0.41^b^
**Api** + **5FU**	1.17 ± 0.41^c^	1.33 ± 0.52^c^
**Se** + **5FU**	1.00 ± 0.00 ^cd^	1.17 ± 0.41^c^
**Api** + **Se** + **5FU**	1.17 ± 0.41^c^	1.17 ± 0.41^c^

*Note:* a‐cd: *p *< 0.001; a‐b, a‐c, b‐c, b‐cd: *p *< 0.01.

### 3.3 Immunohistochemical Results

Immunohistochemical staining performed to assess the expression of Caspase‐3, Bax, and Bcl‐2 revealed significant differences among the experimental groups. Caspase‐3 and Bax immunopositivity in the myocardium was highest in the 5FU group. Caspase‐3 and Bax positive were moderate in the Api + 5FU group, whereas Caspase‐3 was moderate and Bax was mild in the Se + 5FU group. The levels of positive in the Api + Se + 5FU group were mild. Bcl‐2 immunopositivity was severe in the control group, moderate in the Api + 5FU group, and mild in the Se + 5FU and Api + Se + 5FU groups. No significant immunopositivity was observed in the 5FU group (Table 3).

**Table 3:** Statistical evaluation of immunohistochemical findings in myocardium.

Groups	Caspase‐3	Bax	Bcl‐2
**C**	0.17 ± 0.41^a^	0.17 ± 0.41^a^	2.67 ± 0.52^a^
**5FU**	2.83 ± 0.41^b^	2.67 ± 0.52^b^	0.33 ± 0.52^b^
**Api** + **5FU**	2.17 ± 0.41^c^	1.83 ± 0.41^c^	2.00 ± 0.00^e^
**Se** + **5FU**	2.33 ± 0.52^bc^	1.00 ± 0.00^de^	1.17 ± 0.41^c^
**Api** + **Se** + **5FU**	1.33 ± 0.52 ^d^	1.17 ± 0.41 ^d^	1.33 ± 0.52 ^cd^

*Note:* a‐b, a‐bc, a‐c, a‐cd, a‐d, a‐de, b‐d, b‐de, b‐e, c‐de, c‐e: *p *< 0.01; a‐e, b‐c, b‐cd, bc‐d, c‐d, cd‐e: *p *< 0.05.

**Figure 3:**
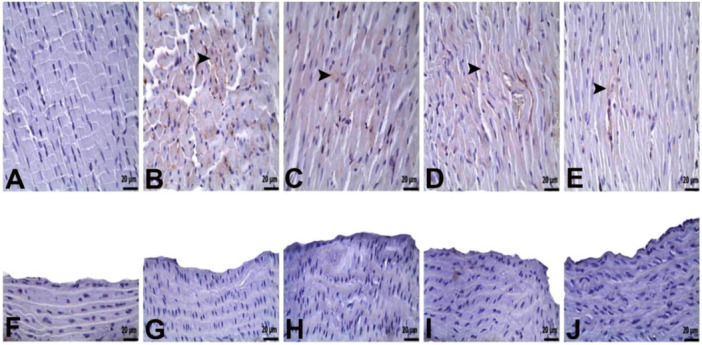
Caspase‐3 immunopositivity in (A–E): Myocardium, (A): Control group. Immune negativity, (B): 5FU group. Severe immunopositivity, (C): Api + 5FU group. Moderate immunopositivity, (D): Se + + 5FU group. Moderate immunopositivity, (E): Api + Se + 5FU group. Mild immunopositivity (arrowhead), (F–J): Caspase‐3 immune negativities in the aorta, (F): Control group, (G): 5FU group, (H): Api + 5FU group, (I): Se + 5FU group, and (J): Api + Se + 5FU group. Immune negativity, IHC.

**Figure 4:**
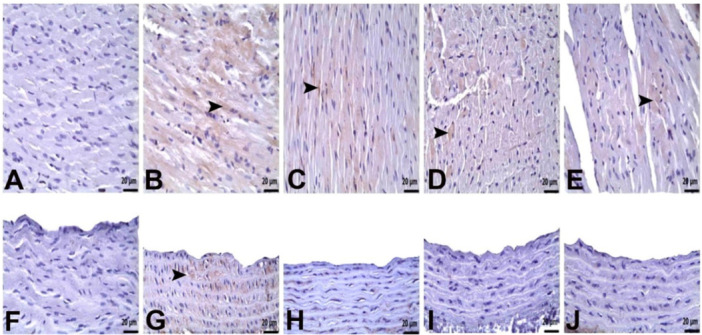
(A–E): Bax immunopositivity in myocardium, (A): Control group. Immune negativity, (B): 5FU group. Severe immunopositivity, (C): Api + 5FU group. Moderate immunopositivity, (D): Se + 5FU group. Mild immunopositivity, (E): Api + Se + 5FU group. Mild immunopositivity (arrowhead), (F–J): Bax immune negativities in the aorta, (F): Control group, Immune negativity (G): 5FU group, Mild immunopositivity, (H): Api + 5FU group, (I): Se + 5FU group, and (J): Api + Se + 5FU group. Immune negativity, IHC.

**Figure 5:**
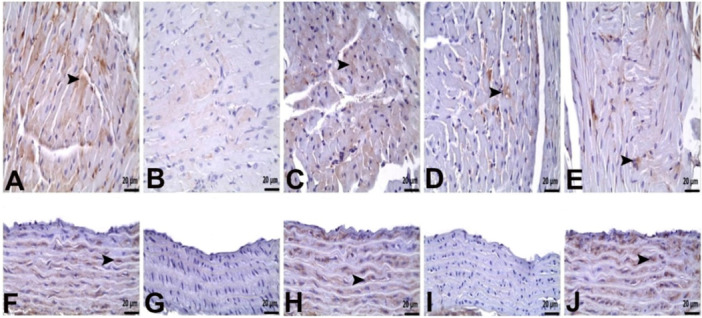
(A–E): Bcl‐2 immunopositivity in myocardium, (A): Control group. Severe immunopositivity, (B): 5FU group. Immune negativity, (C): Api + 5FU group. Moderate immunopositivity, (D): Se + 5FU group. Mild immunopositivity, (E): Api + Se + 5FU group. Mild immunopositivity (arrowhead), (F–J): Bcl‐2 Immune positivity in the aorta, (F): Control group. Moderate immunopositivity, (G): 5FU group. Immune negativity, (H): Api + 5FU group. Moderate immunopositivity, (I): Se + 5FU group. Immune negativity and (J): Api + Se + 5FU group. Moderate immunopositivity, IHC.

Caspase‐3 immunopositivity was not detected in immunohistochemical staining of the aorta, but Bax immunopositivity was determined exclusively in the 5FU group and at a low level. Bcl‐2 immunopositivity was observed at a mild level in the C, Api + 5FU, and Api + Se + 5FU groups, while no significant positivity was observed in the 5FU and Se + 5FU groups (Table 4, Figures 3, 4, 5).

**Table 4:** Statistical evaluation of immunohistochemical findings in the thoracic aorta.

Groups	Caspase‐3	Bax	Bcl‐2
**C**	0.00 ± 0.00	0.00 ± 0.00^a^	1.83 ± 0.41^a^
**5FU**	0.00 ± 0.00	0.67 ± 0.52^b^	0.33 ± 0.52^c^
**Api** + **5FU**	0.00 ± 0.00	0.00 ± 0.00^a^	1.67 ± 0.52^a^
**Se** + **5FU**	0.00 ± 0.00	0.00 ± 0.00^a^	0.33 ± 0.52^c^
**Api** + **Se** + **5FU**	0.00 ± 0.00	0.00 ± 0.00^a^	1.67 ± 0.52^a^

*Note:* a‐c: *p *< 0.01; a‐b: *p *< 0.05.

### 3.4 Biochemical Results

**Figure 6:**
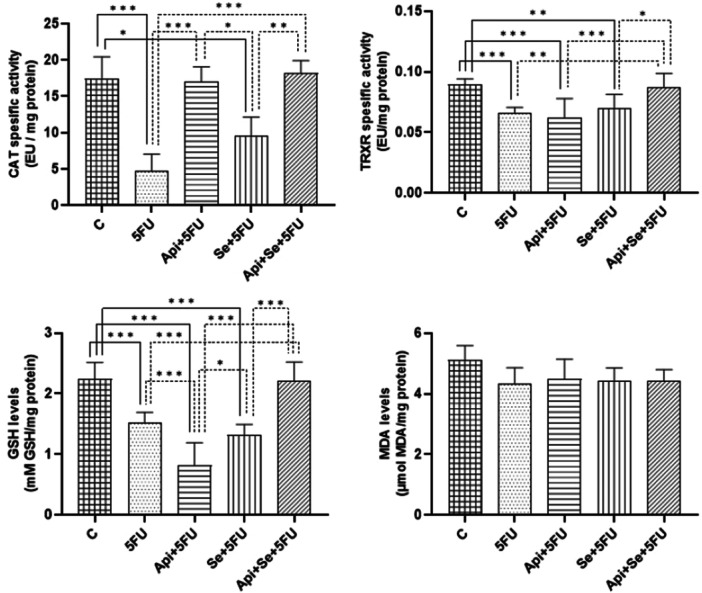
CAT and TrxR activities and GSH and MDA levels in heart tissue of control and experimental groups. *p < 0.05, **p < 0.01, ***p < 0.001.

**Figure 7:**
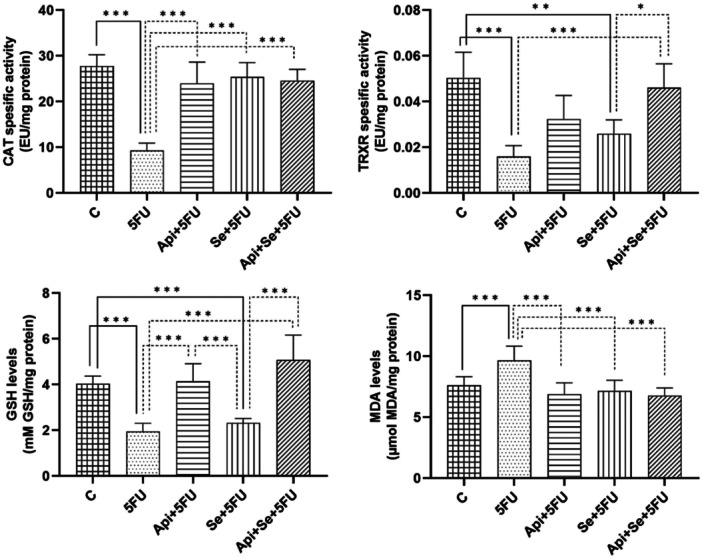
CAT and TrxR activities and GSH and MDA levels in thoracic aorta tissue of control and experimental groups. *p < 0.05, **p < 0.01, ***p < 0.001.

Compared with the control group, the 5FU group exhibited significantly lower GSH levels and CAT and TrxR activities in cardiac tissue (*p* < 0.001). CAT (*p* < 0.001) and TrxR (*p* < 0.01) activities and GSH levels (*p* < 0.001) were significantly increased in the Api + Se + 5FU group compared to the 5FU group, while CAT activities were increased and GSH levels were decreased in the Api + 5FU group (*p* < 0.001). There was no significant difference between the groups in MDA levels (Figure 6). In the thoracic aorta, the 5FU group exhibited significantly lower GSH levels and CAT and TrxR activities, but significantly higher MDA levels, compared with the control group (*p* < 0.001). MDA levels were significantly lower in all treatment groups than in the 5FU group (*p* < 0.001). GSH levels of thoracic aorta tissue were significantly increased in the Api+5FU and Api + Se + 5FU groups, TrxR activity in the Api + Se + 5FU group and CAT activity in all experimental groups (*p* < 0.001) (Figure 7).

## 4 Discussion

Nowadays, chemotherapeutic treatment protocols are continuously updated to increase the survival rate in cancer treatment. The main aim of these updates is to reduce, and if possible, eliminate, treatment‐related toxicity and side effects. The side effects of systemic chemotherapy used in cancer treatment are usually severe. Nephrotoxicity, neurotoxicity, hepatotoxicity, lung toxicity and cardiotoxicity are the leading toxicities that are generally severe [28, 29, 30]. Recent studies have shown that natural compounds with anti‐inflammatory and antioxidant properties can be integrated into treatment regimens to prevent and alleviate the unwanted side effects of chemotherapy and improve the patient's quality of life [31, 32]. The data obtained in this study also support this notion. However, considering that the vast majority of data in the literature comes from experimental studies, it is clear that clinical‐level research is still insufficient and that new research is needed.

The data obtained in the study showed that 5FU caused decreased antioxidant enzyme activity in addition to increased oxidative stress in heart and thoracic aortic tissues and also stimulated apoptosis by increasing the level of apoptotic markers and decreasing antiapoptotic markers. In addition, 5FU caused mononuclear cell infiltration, degeneration, and hemorrhage in the heart and degeneration and necrosis in areas of the thoracic aorta. In parallel with all these toxic effects, the fact that the body weight gain of the animals in the 5FU‐treated group was extremely limited is an indication that it also causes developmental disorders. In previous studies, it was stated that selenium administered as a protective agent to rats with cyclophosphamide‐induced cardiotoxicity reduced MDA and creatine kinase myocardial band (CK‐MB) levels and increased GSH levels, and also significantly reduced cardiac damage [33]. In addition, previous research has reported that selenium administration is effective in correcting the oxidant‐antioxidant status in the liver tissue that is impaired due to 5FU toxicity [34] and in alleviating the inflammation that develops in the intestinal mucosa due to 5FU [35]. Research has demonstrated that apigenin, when given to rats at a dose of 50 mg/kg to prevent lung toxicity caused by 5FU, effectively prevents oxidative stress and oxidative DNA damage, reduces inflammation, and has strong antiapoptotic effects [29]. Similarly, it has been reported that apigenin given as a protector in a model of cisplatin‐induced nephrotoxicity, another chemotherapeutic agent, increases serum antioxidant levels, decreases oxidant levels, relieves inflammation, and shows antiapoptotic effects [36]. It is emphasized that the effects of apigenin may be stronger when applied in combination with other natural resources [29, 36].

5FU is the second most common cytotoxic chemotherapeutic agent associated with cardiotoxicity after anthracyclines [37, 38]. Although it is the most accepted idea that 5FU exerts its cardiotoxicity by inducing myocardial ischemia by causing coronary vasospasm, it is currently thought that its cardiotoxic effect is more extensive than this [39]. Experimental studies have shown that 5‐FU induces severe oxidative stress in cardiac tissue by decreasing GSH levels and the activities of antioxidant enzymes, including SOD, CAT, GPx, and glutathione reductase (GR), while increasing MDA, H_2_O_2_, NO, and lipid peroxide levels [8, 40]. Lokman et al. [41] reported that 5FU administered to rats at a dose of 30 mg/kg for 5 days caused an increase in the levels of oxidants such as MDA and NO, but on the contrary, depletion of antioxidants such as SOD, CAT, GPx, and GSH. In the same study, CK and troponin levels, which are important markers of heart damage, were also shown to be significantly increased. In another study on rats, it was shown that 5FU at a dose of 100 mg/kg administered i.p. increased CK‐MB levels in addition to oxidative stress in cardiac tissue while decreasing total antioxidant levels and vascular endothelial growth factor (VEGF) levels, a potent angiogenic factor [10]. 5FU causes vascular calcification in the smooth muscle cells of rat aortic tissue, according to studies [42]. The findings of the present study demonstrated that 5FU administered at a dose of 150 mg/kg markedly reduced CAT and TrxR activities and GSH levels in both cardiac and thoracic aortic tissues. Moreover, MDA levels were significantly elevated in the thoracic aorta, while no significant alterations in cardiac MDA levels were detected among the experimental groups. Conversely, CAT and TrxR activities together with GSH levels in both cardiac and thoracic aortic tissues were markedly elevated, especially in the apigenin‐plus‐selenium treatment groups, while thoracic aortic MDA levels were significantly reduced.

The most widely accepted mechanism of toxicity of 5FU is that oxidative stress induced due to increased formation of reactive oxygen (ROS) and nitrogen (RNS) species leads to cell membrane damage, increased inflammation, and ultimately stimulation of the apoptotic cell death pathway [40]. Studies have shown that 5FU metabolites such as fluoroacetate and fluorocitrate cause increased oxygen consumption in cardiomyocytes, impair oxidative phosphorylation, increase citrate levels, and deplete high‐energy phosphate compounds, ultimately leading to ATP depletion [43, 44]. Increased oxidative stress in cardiomyocytes and decreased aerobic metabolism due to oxygen depletion cause inflammation and apoptosis in myocardial tissues [45].

It has been reported that administration of 5‐FU at a dose of 150 mg/kg in mice induces myocardial injury, characterized by decreased Bcl‐2 expression and increased Bax and caspase‐3 expression [46]. The results of a previous study showed that 5FU administered to rats at a dose of 30 mg/kg for 5 days induced cardiac apoptosis by decreasing Bcl‐2 expression and increasing caspase‐3 expression [41]. Li et al. [47] reported that mitochondrial membrane potential was disrupted and myocardial fibrosis was observed in rats administered 5FU at a dose of 25 mg/kg for 1 and 2 weeks. In the same study, Western blot analysis revealed that anti‐apoptotic Bcl‐2 levels were decreased, whereas pro‐apoptotic Bax levels were increased in the cardiomyocytes of 5‐FU‐treated rats, leading to the induction of cardiomyocyte apoptosis. In light of the findings of the present study, immunohistochemical analysis demonstrated that 5‐FU induced apoptosis by increasing caspase‐3 and Bax expression and decreasing Bcl‐2 expression in cardiac tissue, while increasing Bax expression and decreasing Bcl‐2 expression in thoracic aortic tissue. In contrast, pretreatment with apigenin, selenium, and their combinations attenuated these effects, as evidenced by decreased pro‐apoptotic caspase‐3 and Bax expression and increased anti‐apoptotic Bcl‐2 expression. These findings further suggest that 5‐FU exerts more pronounced effects on cardiac tissue than on thoracic aortic tissue.

Many studies have shown that 5FU administered to rats at doses of 100 and 125 mg/kg caused high levels of intoxication, severe hyperemia, hyalinization, and necrosis in the heart [8, 10, 48]. The results of the present study showed that 5FU toxicity caused mononuclear cell infiltrations, monocyte degenerations, and myocardial hemorrhages in heart tissue. Degeneration and pyknotic nuclei formations were observed in the thoracic aortic tissue. On the other hand, these findings were considerably attenuated in the groups in which apigenin, selenium, and both substances were administered in combination. It is possible to say that the combined administration of these two substances is more effective in alleviating 5FU‐induced findings.

## 5 Conclusion

Based on the data obtained in this study, we suggest that 5FU causes inflammation by inducing significant histopathological changes in the heart and thoracic aorta tissues, oxidative damage by disrupting the oxidant‐antioxidant balance, severe tissue damage by inducing cellular apoptosis, and ultimately developmental regression. In this study, it was also determined that apigenin and selenium used before 5FU exposure significantly reduced the occurrence of these adverse effects and provided significant cardiovascular protection. The best cardiovascular protection against 5FU toxicity was achieved when both agents were administered together. In the light of these data, we believe that apigenin and selenium are important resources that can be used separately or in combination to prevent and reduce chemotherapy‐induced cardiovascular damage.

## Author Contributions

**Hamit Uslu:** conceptualization, methodology, software, validation, formal analysis, investigation, resources, data curation, writing – original draft, writing – review and editing, visualization, supervision, project administration. **Gözde Atila Uslu:** conceptualization, methodology, software, validation, formal analysis, investigation, resources, data curation, writing – original draft, writing – review and editing, visualization, supervision, project administration. **Taha Abdulkadir Çoban:** conceptualization, methodology, investigation, resources, writing – review and editing. **Emine Toraman:** methodology, software, investigation, resources, data curation, writing – original draft. **Mustafa Özkaraca:** methodology, software, resources, data curation, visualization.

## Funding

The authors have nothing to report.

## Ethics Statement

All experimental procedures performed on experimental animals were performed in accordance with national and international guidelines. The necessary permissions for this study were obtained from Erzincan Binali Yıldırım University Animal Experiments Local Ethics Committee (protocol number: 2024/42).

## Conflicts of Interest

The authors declare no conflicts of interest.

## Data Availability

The data that support the findings of this study are available on request from the corresponding author. The data are not publicly available due to privacy or ethical restrictions.
